# 1703. Efficacy of Selected Metabolic Inhibitors for the Prevention and Resensitization of High-Level Daptomycin Resistance in *Streptococcus mitis-oralis In Vitro* and in an *Ex Vivo* Simulated Endocarditis Model

**DOI:** 10.1093/ofid/ofac492.1333

**Published:** 2022-12-15

**Authors:** Razieh Kebriaei, Michael J Rybak, Christian K Lapitan, Greg A Somerville, Arnold S Bayer, Nagendra N Mishra

**Affiliations:** Eugene Applebaum College of Pharmacy and Health Sciences, Detroit, Michigan; Eugene Applebaum College of Pharmacy and Health Sciences, Detroit, Michigan; Lundquist Institute-Harbor UCLA Medical Center, Torrance, California; School of Veterinary Medicine and Biomedical Sciences, University of Nebraska-Lincoln, Lincoln, Nebraska; Lundquist Institute-Harbor UCLA Medical Center, Torrance, California; Lundquist Institute-Harbor UCLA Medical Center, Torrance, California

## Abstract

**Background:**

Rapid development of stable, high-level daptomycin-resistance (DAP-R) is frequent among *Streptococcus mitis-oralis* strains during exposure to DAP *in vitro* and *in vivo*. Metabolomic analyses of *in vitro*-derived daptomycin-resistant (DAP-R) *S. mitis-oralis* strains (351D10) revealed substantial glycolytic pathway differences vs. its DAP-susceptible (DAP-S) parental strain, 351. To define translatability of such metabolic changes, we assessed combinations of DAP + strategic metabolic inhibitors likely to impact such pathways, including: oxamic acid [OXA], trimetazidine [TMZ) and 6-mercaptopurine [6-MP]. We assessed these combinations vs. our DAP-S/DAP-R isogenic strain pair both *in vitro* and *ex vivo* for: bactericidal and synergistic activities; prevention of high-level DAP-R in DAP-S cells; and/or resensitization of DAP-R cells

**Methods:**

**MICs.** E test and microbroth dilution

***In vitro* antimicrobial combination assays.** Time-kill curves (24 hr) and serial passaging in DAP (10 d).

***Ex vivo* IE model:** SEVs (simulated endocardial vegetation’s) were quantitatively cultured at serial time-points post-exposures to DAP +/- inhibitors (0, 4, 8, 24, 32, 48 hr).

**Results:**

*In vitro*, combinations of DAP + OXA or DAP + TMZ, as well as OXA or TMZ monotherapy, exerted bactericidal effect against both strains. Moreover, DAP + 6-MP yielded both a bactericidal, as well as synergistic activity against DAP-S 351 (but not against DAP-R 351 D10). None of the combinations prevented the development of DAP-R or resensitized the DAP-R strain to a DAP-S phenotype *in vitro*. In the *ex vivo* SEV model, using PK/PD simulations of humanized drug doses, DAP + OXA was the most effective regimen for both the DAP-S 351 and DAP-R 351 D10 strains; both OXA alone and DAP + OXA prevented DAP-R evolution, although not resensitizing the DAP-R strain to a DAP-S phenotype.

**Conclusion:**

Combinations of DAP plus specific metabolic inhibitors represents a promising approach to enhance killing of *S. mitis-oralis* strains, as well as to potentially forestall DAP-R emergence. Also, they do provide a solid platform upon which to explore other potential metabolic modifiers for their ability to enhance the efficacy of DAP, based on definable metabolic perturbations in DAP-R *S. mitis-oralis.*

**Disclosures:**

**All Authors**: No reported disclosures.

